# A Sample to Results Workflow for Compositional Analysis of Multiplexed Amplicon Sequencing Experiments

**DOI:** 10.64898/2026.07.28.741237

**Published:** 2026-07-28

**Authors:** Alexa Bennett, Ryan Moore, Craig W. Herbold, Thomas E. Hanson

## Abstract

Microbial communities play key roles in the transformation and cycling of elements ranging from required macronutrients to toxic metalloids. Next-generation sequencing has been applied across multiple ecosystems to probe the interplay of microbial community structure and functional potential with respect to elemental cycling. Shotgun metagenomics collects marker gene sequences without amplification and is costly for large numbers of samples and deep coverage. Conversely, amplicon sequencing of taxonomic marker genes, e.g. 16S and 18S rRNA, is cost-effective for large numbers of samples, but provides limited functional insight. A middle ground between the two approaches is needed to analyze community structure and functional potential within a sample while remaining cost-effective with high throughput. To address this need, we developed a standardized workflow for multiplexed amplicon sequencing from sample collection through data analysis for diverse sample types, including freshwater, sediments, and soils, that produces data and publication-ready figures for multiple taxonomic and functional genes for carbon, nitrogen, phosphorus, sulfur, and arsenic cycling for each sample analyzed. The workflow’s utility was shown by analyzing 11 taxonomic and functional gene amplicons sequenced from 25 samples with high technical replicate similarity. The workflow is named *CAMASE* for *C* ompositional *A* nalysis of *M* ultiplex *A* mplicon *S* equencing *E* xperiments. This proof-of-concept shows that CAMASE economically produces standard amplicon sequencing outputs (ASV/OTU counts and taxonomy, PCA, and relative abundance plots) for hundreds of amplicon by sample combinations and provides specific recommendations for implementation.

**GRAPHICAL ABSTRACT:**

Samples are collected in a preservative and material collected on filters prior to DNA extraction. Target gene amplicons are produced in parallel with internal barcodes enabling sequencing in a single run followed by compositional data analysis. All wet lab protocols, code markdowns, and templates for required metadata files are available at https://hansonlabgit.dbi.udel.edu/aprange/CAMASE . Created in BioRender. Bennett, A. (2026) https://BioRender.com/ymnojt0

## Full Text Availability

The license terms selected by the author(s) for this preprint version do not permit archiving in PMC. The full text is available from the preprint server.

